# 6,6′-Dieth­oxy-2,2′-[hexane-1,6-diylbis(nitrilo­methanylyl­idene)]diphenol

**DOI:** 10.1107/S1600536811009445

**Published:** 2011-03-19

**Authors:** Kwang Ha

**Affiliations:** a School of Applied Chemical Engineering, The Research Institute of Catalysis, Chonnam National University, Gwangju 500-757, Republic of Korea

## Abstract

The title compound, C_24_H_32_N_2_O_4_, is a polydentate Schiff base and reveals strong intra­molecular O—H⋯N hydrogen bonding between the hy­droxy O atom and the imino N atom, with an O⋯N distance of 2.570 (3) Å. In the crystal, a centre of inversion is located at the mid-point of the compound. The diimino­hexyl­ene chain is almost ideally in the *anti* conformation, with an average dihedral angle of 179.0°.

## Related literature

For related structures, see: Bermejo *et al.* (2007 ▶); Fun *et al.* (2009 ▶); Ha (2010 ▶).
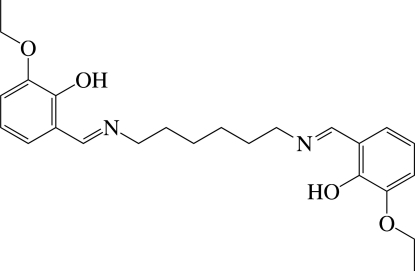


## Experimental

### 

#### Crystal data

C_24_H_32_N_2_O_4_
*M* *_r_* = 412.52
Triclinic, 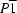

*a* = 6.9094 (13) Å
*b* = 6.9184 (13) Å
*c* = 11.936 (2) Å
α = 91.271 (5)°
β = 99.677 (4)°
γ = 102.550 (4)°
*V* = 547.97 (18) Å^3^
*Z* = 1
Mo *K*α radiation
μ = 0.09 mm^−1^
*T* = 200 K
0.26 × 0.23 × 0.23 mm

#### Data collection

Bruker SMART 1000 CCD diffractometer
Absorption correction: multi-scan (*SADABS*; Bruker, 2000 ▶) *T*_min_ = 0.862, *T*_max_ = 1.000
3505 measured reflections
2140 independent reflections
1258 reflections with *I* > 2σ(*I*)
*R*_int_ = 0.031

#### Refinement

*R*[*F* ^2^ > 2σ(*F* ^2^)] = 0.062
*wR*(*F* ^2^) = 0.168
*S* = 1.02
2140 reflections
138 parameters
H-atom parameters constrained
Δρ_max_ = 0.23 e Å^−3^
Δρ_min_ = −0.21 e Å^−3^

### 

Data collection: *SMART* (Bruker, 2000 ▶); cell refinement: *SAINT* (Bruker, 2000 ▶); data reduction: *SAINT*; program(s) used to solve structure: *SHELXS97* (Sheldrick, 2008 ▶); program(s) used to refine structure: *SHELXL97* (Sheldrick, 2008 ▶); molecular graphics: *ORTEP-3* (Farrugia, 1997 ▶) and *PLATON* (Spek, 2009 ▶); software used to prepare material for publication: *SHELXL97*.

## Supplementary Material

Crystal structure: contains datablocks global, I. DOI: 10.1107/S1600536811009445/ds2098sup1.cif

Structure factors: contains datablocks I. DOI: 10.1107/S1600536811009445/ds2098Isup2.hkl

Additional supplementary materials:  crystallographic information; 3D view; checkCIF report

## Figures and Tables

**Table 1:** Hydrogen-bond geometry (Å, °)

*D*—H⋯*A*	*D*—H	H⋯*A*	*D*⋯*A*	*D*—H⋯*A*
O1—H1⋯N1	0.84	1.83	2.570 (3)	147

**Fig. 1.**
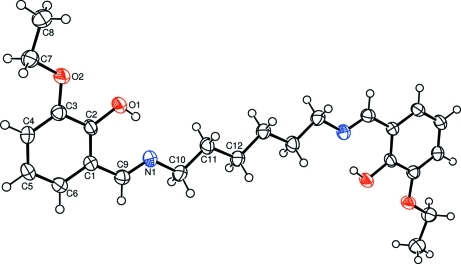
The structure of the title compound, with displacement ellipsoids drawn at the 50% probability level; H atoms are shown as small circles of arbitrary radius. Unlabelled atoms are related to the reference atoms by the (1 - x, 2 - y, 1 - z) symmetry transformation.

**Fig. 2.**
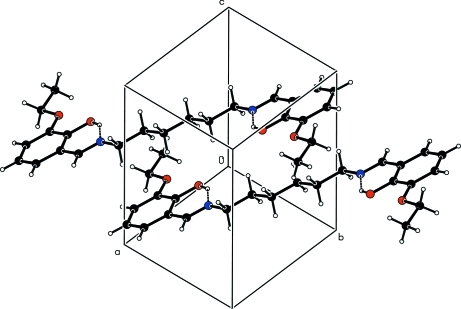
View of the unit-cell contents of the title compound. Hydrogen-bond interactions are drawn with dashed lines.

## supplementary crystallographic information

### Comment

The title compound crystallized in the triclinic space group *P*1, same to the analogous compounds with propylene chain (C_21_H_26_N_2_O_4_) (Ha, 2010) and butylene chain (C_22_H_28_N_2_O_4_) (Fun *et al.*, 2009), whereas the related Schiff base with ethylene group (C_20_H_24_N_2_O_4_) crystallized in the monoclinic space group *C*2/*c* (Bermejo *et al.*, 2007).

The asymmetric unit of the title molecule contains one half of the formula unit; a centre of inversion is located in the midpoint of the compound (Fig. 1). The Schiff base reveals strong intramolecular O—H···N hydrogen bonding between the hydroxy O atom and the imino N atom with d(O···N) = 2.570 (3) Å forming a nearly planar six-membered ring (Fig. 2, Table 1). The N1—C9/10 bond lengths and the C9—N1—C10 bond angle indicate that the imino N1 atom is *sp*^2^-hybridized [*d*(N1═C9) = 1.271 (3) Å and d(N1—C10) = 1.469 (3) Å; <C9—N1—C10 = 121.2 (2)°]. The torsion angles for the four atoms within the diiminohexylene chain indicate that the chain is almost perfectly in the anti conformation with <N1—C10—C11—C12 = 179.1 (2)° and <C10—C11—C12—C12^i^ (symmetry code i: 1 - *x*, 2 - *y*, 1 - *z*) = 178.8 (3)°.

### Experimental

1,6-Diaminohexane (0.8132 g, 6.998 mmol) and 3-ethoxysalicylaldehyde (2.3265 g, 14.000 mmol) in EtOH (20 ml) were stirred for 5 h at room temperature. After addition of pentane (30 ml) to the reaction mixture, the formed precipitate was separated by filtration, washed with ether, and dried at 50 °C, to give a yellow powder (2.3563 g). Crystals suitable for X-ray analysis were obtained by slow evaporation from a toluene solution.

### Refinement

H atoms were positioned geometrically and allowed to ride on their respective parent atoms [C—H = 0.95 Å (CH), 0.99 Å (CH_2_) or 0.98 Å (CH_3_) and O—H = 0.84 Å, and *U*_iso_(H) = 1.2*U*_eq_(C) or 1.5*U*_eq_(methyl C, O)].

### Crystal data

**Table:**

C_24_H_32_N_2_O_4_	*Z* = 1
*M**_r_* = 412.52	*F*(000) = 222
Triclinic, *P*1	*D*_x_ = 1.250 Mg m^−^^3^
Hall symbol: -P 1	Mo *K*α radiation, λ = 0.71073 Å
*a* = 6.9094 (13) Å	Cell parameters from 873 reflections
*b* = 6.9184 (13) Å	θ = 3.1–25.7°
*c* = 11.936 (2) Å	µ = 0.09 mm^−^^1^
α = 91.271 (5)°	*T* = 200 K
β = 99.677 (4)°	Stick, yellow
γ = 102.550 (4)°	0.26 × 0.23 × 0.23 mm
*V* = 547.97 (18) Å^3^	

### Data collection

**Table:**

Bruker SMART 1000 CCD diffractometer	2140 independent reflections
Radiation source: fine-focus sealed tube	1258 reflections with *I* > 2σ(*I*)
graphite	*R*_int_ = 0.031
φ and ω scans	θ_max_ = 26.0°, θ_min_ = 3.0°
Absorption correction: multi-scan (*SADABS*; Bruker, 2000)	*h* = −8→8
*T*_min_ = 0.862, *T*_max_ = 1.000	*k* = −8→8
3505 measured reflections	*l* = −14→11

### Refinement

**Table:**

Refinement on *F*^2^	Primary atom site location: structure-invariant direct methods
Least-squares matrix: full	Secondary atom site location: difference Fourier map
*R*[*F*^2^ > 2σ(*F*^2^)] = 0.062	Hydrogen site location: inferred from neighbouring sites
*wR*(*F*^2^) = 0.168	H-atom parameters constrained
*S* = 1.02	*w* = 1/[σ^2^(*F*_o_^2^) + (0.0725*P*)^2^ + 0.0011*P*] where *P* = (*F*_o_^2^ + 2*F*_c_^2^)/3
2140 reflections	(Δ/σ)_max_ < 0.001
138 parameters	Δρ_max_ = 0.23 e Å^−^^3^
0 restraints	Δρ_min_ = −0.21 e Å^−^^3^

### Special details

**Table:**

Geometry. All e.s.d.'s (except the e.s.d. in the dihedral angle between two l.s. planes) are estimated using the full covariance matrix. The cell e.s.d.'s are taken into account individually in the estimation of e.s.d.'s in distances, angles and torsion angles; correlations between e.s.d.'s in cell parameters are only used when they are defined by crystal symmetry. An approximate (isotropic) treatment of cell e.s.d.'s is used for estimating e.s.d.'s involving l.s. planes.
Refinement. Refinement of *F*^2^ against ALL reflections. The weighted *R*-factor *wR* and goodness of fit *S* are based on *F*^2^, conventional *R*-factors *R* are based on *F*, with *F* set to zero for negative *F*^2^. The threshold expression of *F*^2^ > σ(*F*^2^) is used only for calculating *R*-factors(gt) *etc*. and is not relevant to the choice of reflections for refinement. *R*-factors based on *F*^2^ are statistically about twice as large as those based on *F*, and *R*- factors based on ALL data will be even larger.

### Fractional atomic coordinates and isotropic or equivalent isotropic displacement parameters (Å^2^)

**Table:**

	*x*	*y*	*z*	*U*_iso_*/*U*_eq_	
O1	0.5421 (2)	0.2477 (3)	0.24546 (17)	0.0424 (5)	
H1	0.5714	0.3570	0.2834	0.064*	
O2	0.5003 (2)	−0.0878 (2)	0.13057 (16)	0.0405 (5)	
N1	0.7714 (3)	0.5589 (3)	0.35709 (19)	0.0383 (6)	
C1	0.9039 (3)	0.3153 (3)	0.2687 (2)	0.0312 (6)	
C2	0.7131 (3)	0.1965 (3)	0.2277 (2)	0.0318 (6)	
C3	0.6946 (4)	0.0168 (4)	0.1652 (2)	0.0327 (6)	
C4	0.8654 (4)	−0.0394 (4)	0.1439 (2)	0.0367 (7)	
H4	0.8526	−0.1599	0.1007	0.044*	
C5	1.0562 (4)	0.0782 (4)	0.1849 (2)	0.0399 (7)	
H5	1.1729	0.0374	0.1705	0.048*	
C6	1.0750 (4)	0.2543 (4)	0.2467 (2)	0.0357 (7)	
H6	1.2051	0.3348	0.2745	0.043*	
C7	0.4707 (4)	−0.2781 (3)	0.0726 (2)	0.0400 (7)	
H7A	0.5449	−0.3639	0.1196	0.048*	
H7B	0.5193	−0.2641	−0.0009	0.048*	
C8	0.2475 (4)	−0.3664 (4)	0.0530 (3)	0.0492 (8)	
H8A	0.2003	−0.3725	0.1261	0.074*	
H8B	0.2202	−0.5005	0.0170	0.074*	
H8C	0.1767	−0.2835	0.0032	0.074*	
C9	0.9234 (4)	0.5017 (4)	0.3333 (2)	0.0351 (6)	
H9	1.0540	0.5831	0.3585	0.042*	
C10	0.7949 (4)	0.7477 (4)	0.4222 (2)	0.0394 (7)	
H10A	0.8727	0.8568	0.3846	0.047*	
H10B	0.8707	0.7421	0.4998	0.047*	
C11	0.5920 (4)	0.7884 (3)	0.4300 (2)	0.0384 (7)	
H11A	0.5143	0.6766	0.4657	0.046*	
H11B	0.5179	0.7939	0.3520	0.046*	
C12	0.6021 (3)	0.9794 (4)	0.4974 (2)	0.0366 (7)	
H12A	0.6735	0.9729	0.5760	0.044*	
H12B	0.6819	1.0911	0.4626	0.044*	

### Atomic displacement parameters (Å^2^)

**Table:**

	*U*^11^	*U*^22^	*U*^33^	*U*^12^	*U*^13^	*U*^23^
O1	0.0309 (10)	0.0439 (11)	0.0537 (13)	0.0112 (8)	0.0094 (9)	−0.0110 (9)
O2	0.0304 (10)	0.0369 (10)	0.0523 (13)	0.0049 (7)	0.0070 (9)	−0.0091 (9)
N1	0.0373 (12)	0.0389 (13)	0.0416 (14)	0.0150 (10)	0.0080 (11)	−0.0040 (11)
C1	0.0289 (13)	0.0342 (14)	0.0323 (15)	0.0099 (10)	0.0068 (12)	0.0001 (12)
C2	0.0278 (13)	0.0360 (14)	0.0356 (16)	0.0128 (11)	0.0096 (12)	0.0012 (12)
C3	0.0330 (14)	0.0323 (14)	0.0331 (15)	0.0076 (11)	0.0061 (12)	0.0017 (12)
C4	0.0387 (14)	0.0367 (14)	0.0372 (16)	0.0129 (11)	0.0087 (13)	−0.0053 (12)
C5	0.0318 (14)	0.0461 (16)	0.0465 (18)	0.0148 (12)	0.0127 (13)	−0.0029 (14)
C6	0.0282 (13)	0.0389 (15)	0.0401 (16)	0.0075 (11)	0.0066 (12)	−0.0007 (12)
C7	0.0397 (15)	0.0322 (15)	0.0469 (18)	0.0078 (11)	0.0052 (13)	−0.0025 (13)
C8	0.0450 (16)	0.0375 (16)	0.060 (2)	−0.0003 (12)	0.0075 (15)	−0.0070 (14)
C9	0.0305 (13)	0.0347 (14)	0.0387 (16)	0.0049 (11)	0.0056 (12)	−0.0011 (12)
C10	0.0410 (15)	0.0387 (15)	0.0385 (17)	0.0123 (12)	0.0037 (13)	−0.0083 (13)
C11	0.0401 (15)	0.0351 (15)	0.0429 (17)	0.0121 (11)	0.0112 (13)	−0.0035 (13)
C12	0.0381 (15)	0.0336 (15)	0.0387 (16)	0.0107 (11)	0.0056 (13)	−0.0032 (12)

### Geometric parameters (Å, °)

**Table:**

O1—C2	1.353 (3)	C7—C8	1.506 (4)
O1—H1	0.8400	C7—H7A	0.9900
O2—C3	1.369 (3)	C7—H7B	0.9900
O2—C7	1.431 (3)	C8—H8A	0.9800
N1—C9	1.271 (3)	C8—H8B	0.9800
N1—C10	1.469 (3)	C8—H8C	0.9800
C1—C2	1.396 (3)	C9—H9	0.9500
C1—C6	1.401 (3)	C10—C11	1.506 (3)
C1—C9	1.456 (3)	C10—H10A	0.9900
C2—C3	1.406 (3)	C10—H10B	0.9900
C3—C4	1.379 (3)	C11—C12	1.514 (3)
C4—C5	1.393 (3)	C11—H11A	0.9900
C4—H4	0.9500	C11—H11B	0.9900
C5—C6	1.380 (3)	C12—C12^i^	1.510 (4)
C5—H5	0.9500	C12—H12A	0.9900
C6—H6	0.9500	C12—H12B	0.9900
			
C2—O1—H1	109.5	C7—C8—H8A	109.5
C3—O2—C7	117.46 (18)	C7—C8—H8B	109.5
C9—N1—C10	121.2 (2)	H8A—C8—H8B	109.5
C2—C1—C6	119.5 (2)	C7—C8—H8C	109.5
C2—C1—C9	119.9 (2)	H8A—C8—H8C	109.5
C6—C1—C9	120.6 (2)	H8B—C8—H8C	109.5
O1—C2—C1	122.4 (2)	N1—C9—C1	122.2 (2)
O1—C2—C3	117.9 (2)	N1—C9—H9	118.9
C1—C2—C3	119.8 (2)	C1—C9—H9	118.9
O2—C3—C4	126.0 (2)	N1—C10—C11	110.5 (2)
O2—C3—C2	114.4 (2)	N1—C10—H10A	109.6
C4—C3—C2	119.6 (2)	C11—C10—H10A	109.6
C3—C4—C5	120.9 (2)	N1—C10—H10B	109.6
C3—C4—H4	119.6	C11—C10—H10B	109.6
C5—C4—H4	119.6	H10A—C10—H10B	108.1
C6—C5—C4	119.7 (2)	C10—C11—C12	114.0 (2)
C6—C5—H5	120.2	C10—C11—H11A	108.7
C4—C5—H5	120.2	C12—C11—H11A	108.7
C5—C6—C1	120.6 (2)	C10—C11—H11B	108.7
C5—C6—H6	119.7	C12—C11—H11B	108.7
C1—C6—H6	119.7	H11A—C11—H11B	107.6
O2—C7—C8	106.4 (2)	C12^i^—C12—C11	113.5 (3)
O2—C7—H7A	110.4	C12^i^—C12—H12A	108.9
C8—C7—H7A	110.4	C11—C12—H12A	108.9
O2—C7—H7B	110.4	C12^i^—C12—H12B	108.9
C8—C7—H7B	110.4	C11—C12—H12B	108.9
H7A—C7—H7B	108.6	H12A—C12—H12B	107.7
			
C6—C1—C2—O1	−179.6 (2)	C3—C4—C5—C6	−0.7 (4)
C9—C1—C2—O1	0.1 (4)	C4—C5—C6—C1	0.2 (4)
C6—C1—C2—C3	0.2 (4)	C2—C1—C6—C5	0.0 (4)
C9—C1—C2—C3	179.9 (2)	C9—C1—C6—C5	−179.6 (3)
C7—O2—C3—C4	3.8 (4)	C3—O2—C7—C8	175.5 (2)
C7—O2—C3—C2	−176.2 (2)	C10—N1—C9—C1	−179.9 (3)
O1—C2—C3—O2	−0.9 (4)	C2—C1—C9—N1	2.1 (4)
C1—C2—C3—O2	179.3 (2)	C6—C1—C9—N1	−178.3 (3)
O1—C2—C3—C4	179.1 (2)	C9—N1—C10—C11	175.2 (3)
C1—C2—C3—C4	−0.7 (4)	N1—C10—C11—C12	179.1 (2)
O2—C3—C4—C5	−179.1 (2)	C10—C11—C12—C12^i^	178.8 (3)
C2—C3—C4—C5	0.9 (4)		

Symmetry codes: (i) −*x*+1, −*y*+2, −*z*+1.

### Hydrogen-bond geometry (Å, °)

**Table:**

*D*—H···*A*	*D*—H	H···*A*	*D*···*A*	*D*—H···*A*
O1—H1···N1	0.84	1.83	2.570 (3)	147
